# An American Text-Book of Pathology

**Published:** 1902-02

**Authors:** 


An American Text-Book of Pathology. Edited by Ludvig Hektoen, M. D., Professor of Pathology, Rush Medical College, Chicago; and David Riesman, M. D., Professor of Clinical Medicine, Philadelphia Polyclinic. Handsome imperial 8vo of 1245 pages, 443 illustrations, 66 of them in colors. Philadelphia and London: W. B. Saunders & Co. 1901. [Cloth, $7.50; sheep or half-morocco, $8.50 net.]

The above work is the first attempt to produce a truly American text-book of pathology. It has the disadvantage that so many of the recent American books possess, of being composed of chapters furnished by different contributors (in this case nineteen.) For this reason the chapters show great variation in the treatment of the different subjects and the book, as a whole, suffers from that lack of coherence which marks all work of this class. Otherwise the work makes an excellent appearance and will undoubtedly reflect credit upon American pathology.

The illustrations are largely new, a great many of them excellent; at the same time it is to be regretted that in a work of this kind, any illustrations should be borrowed from foreign text-books, and it is to be hoped that in the future editions of the work, such may be replaced by original illustrations. Chapter I. on Health and Disease contains a great deal of suggestive material. The chapter on general morbid processes is carefully prepared and clearly written. The chapter on tumors, considering the present status of the question, is clear. The classification is that generally accepted by pathologists. The placing of glioma and neuroma after the fibro-epithelial tumors instead of between them and the strictly connective tissue ones, somewhat breaks the symmetry and the broad classification into non-malignant and malignant tumors is more in the interest of clinical pathology than of strict classification. The chapter on micro-parasites is sufficiently exhaustive for a text-book on pathology. The space allotted to the budding and filamentous fungi, especially an excellent chapter on pathogenic protozoa bear evidence of the growing interest in these organisms. The chapter on animal parasites is concise and clear. The intoxications, epescially the mineral poisons, receive somewhat fuller treatment than is customary. The chapter on fever is concise and that on teratology is accompanied by a number of excellent illustrations.

About two-thirds of the book is devoted to special pathology and contains a series of excellent chapters. It is interesting to note the introduction of a special chapter on the blood by a recognised hematologist. The chapter on diseases of the central nervous system, contains several original colored illustrations of great excellence. The chapter upon the bones is perhaps a little too brief for so important a subject. The illustrations are mostly original and generally good. In the chapter on the voluntary muscles, the illustrations fall far below the general average of excellence. The text of the chapter on the digestive system is excellent, but the illustrations with two exceptions are inferior.

There are several photomicrographs, notably those on pages 762, 790, 802, 806, 810, 813, 814, 816 and 823, which mar the appearance of the chapter and should be replaced either by suitable drawings or properly reproduced photomicrographs from good negatives. The chapter on the respiratory tract is not as exhaustive as could be wished. The illustrations are generally good; several of the colored ones excellent. The chapter on the ductless glands, considering the limited knowledge of this subject is treated in a very able manner. The chapter on the urinary organs follows accepted lines. In the classification of inflammations of the kidney an attempt is made to combine the clinical and pathological classifications. The illustrations of histological preparations are generally good. Those of the macroscopic specimens of the kidney are generally too highly colored. The chapter on the female genital tract is excellent, and accompanied by a number of good drawings. It is rather a curious innovation to treat diseases of the breast under a separate heading. The chapter is ably written and well illustrated, containing a number of photomicrographs satisfactorily reproduced; at the same time, the propriety of according separate treatment to this organ may be questioned. The chapters on diseases of the skin and the eye and ear are sufficiently exhaustive and well illustrated.

On the whole the book makes a most satisfactory impression and can be warmly welcomed as the first attempt to produce an exhaustive American text-book of pathology. The introduction of the colored illustrations in the text adds greatly to the appearance of the work and is a pleasing characteristic. The work is worthy of the support of all the American teachers and satisfactorialy represents the present-day status of American pathology. —H. R. G.

